# Suppression of Inflammation, Osteoclastogenesis and Bone Loss by PZRAS Extract

**DOI:** 10.4014/jmb.2004.04016

**Published:** 2020-08-15

**Authors:** Liang Li, Young-Ran Park, Saroj Kumar Shrestha, Hyoung-Kwon Cho, Yunjo Soh

**Affiliations:** 1 Department of Dental Pharmacology, School of Dentistry, Jeonbuk National University, Jeonju 54896, Republic of Korea; 2 Hanpoong Pharm and Foods Co., Ltd., Jeonju 561-841, Republic of Korea; 3 Department of Pharmacology, School of Pharmacy and Institute of New Drug Development, Jeonbuk National University, Jeonju 54896, Republic of Korea

**Keywords:** PZRAS, antioxidant, anti-inflammatory, osteoclastogenesis, bone loss

## Abstract

*Panax ginseng* has a wide range of activities including a neuroprotective effect, skin protective effects, enhanced DNA repairing, anti-diabetic activity, and protective effects against vascular inflammation. In the present study, we sought to discover the inhibitory effects of a mixture of natural products containing *Panax ginseng*, *Ziziphus jujube*, *Rubi fructus*, *Artemisiae asiaticae* and *Scutellaria baicalensis* (PZRAS) on osteoclastogenesis and bone remodeling, as neither the effects of a mixture containing *Panax ginseng* extract, nor its molecular mechanism on bone inflammation, have been clarified yet. PZRAS upregulated the levels of catalase (CAT), superoxide dismutase (SOD), glutathione reductase (GSH-R) and glutathione peroxidase (GSH-Px) and reduced malondialdehyde (MDA) in LPS-treated RAW264.7 cells. Moreover, treatment with PZRAS decreased the production of IL-1β and TNF-α. PZRAS also inhibited osteoclast differentiation through inhibiting osteoclastspecific genes like MMP-2, 9, cathepsin K, and TRAP in RANKL-treated RAW264.7 cells. Additionally, PZRAS has inhibitory functions on the RANKL-stimulated activation of ERK and JNK, which lead to a decrease in the expression of NFATc1 and c-Fos. In an in vivo study, bone resorption induced by LPS was recovered by treatment with PZRAS in bone volume per tissue volume (BV/TV) compared to control. Furthermore, the ratio of eroded bone surface of femurs was significantly increased in LPStreated mice compared to vehicle group, but this ratio was significantly reversed in PZRAS-treated mice. These results suggest that PZRAS could prevent or treat disorders with abnormal bone loss.

## Introduction

Osteoporosis, characterized by low bone mass and destruction of bone microarchitecture, is a complicated disorder with multiple factors including estrogen deficiency, the aging process, and genetics, and leads to fracture risk predominantly in the older population [1]. Anti-bone resorptive drugs such as bisphosphonates, with their excellent bone-improving effects, have been extensively used [2]. However, many previous studies have reported that some of these drugs are also associated with various diseases including endo-metritis, thromboembolism, breast cancer, hypercalcemia, atrial fibrillation, and osteonecrosis of the jaw [3-5].

Natural flavonoid compounds are good resources for therapeutic agents because they do not carry severe risk along with the possibility of long-term treatment. Therefore, the selection of natural compounds is important in developing drugs for osteoporosis [7]. Many plants have been incorporated into domestic medicines and have anti-inflammatory and pharmacological properties ascribed to antioxidant compounds [6, 8].

During inflammation, reactive oxygen species (ROS) are produced to defend against infection [9,10]. Especially, excessive nitric oxide (NO) abnormally released by inducible nitric oxide synthase (iNOS) can damage macromolecules such as proteins, DNA, and lipids [11]. In recent reports, free radicals were found to be elevated in response to osteoclastic activity and lipid peroxidation [12]. In addition, the reduction of the antioxidant condition was manifested by low levels of glutathione peroxidase (GSH-Px), glutathione reductase (GSH-R) and superoxide dismutase (SOD) [13]. Matrix metalloproteinases (MMPs) are a group of proteinase-related enzymes that degrade the extracellular matrix. Increased production of MMPs is associated with chronic inflammatory diseases such as rheumatoid arthritis and periodontitis [14-18].

Some recent studies have reported that lipopolysaccharide (LPS)-stimulated osteoclastogenesis exacerbates bone destruction [19, 20]. LPS plays an essential role in bone resorption, which includes assembling inflammatory cells, production of cytokines such as interleukin-1β (IL-1β) and tumor necrosis factor- α (TNF-α), and induction of osteoclastic differentiation [21]. Therefore, it is a beneficial approach to treat various disorders by reducing oxidative stress and inflammation processes.

*Panax ginseng* is a traditional herbal medicine employed as a general health-promoting tonic. Ginsenoside-Rg2 is the main active component that can be extracted from the root and stem leaves of *P. ginseng*. Additionally, ginsenoside-Rg2 has been known to act through a variety of mechanisms to mediate a neuroprotective effect, a skin protective effect, enhanced DNA repair, anti-diabetic activity and a protective effect against vascular inflammation [22, 23]. *Ziziphus jujube* has been used as herbal medicine and to cure oxidative stress-related diseases such as tumors and cardiovascular diseases [24]. Recently, goshonoside-F5 from *Rubi fructus* was reported to inhibit the Nuclear factor-κappa B (NF-κB) and MAPK signaling pathways [25]. The extract of *Astragalus membranaceus* has displayed anti-inflammatory and antioxidant effects in vitro [26]. Some compounds from *Artemisia argyi* supply a new lead compound for inflammatory disorders [27]. *Z. jujube* and *Scutellaria baicalensis* were known to have potential bone-forming effects [28]. However, there has been little investigation into the effect of mixed medicinal plant extracts containing *P. ginseng* on bone lytic disorders.

Here, we explored the antioxidant, anti-inflammatory, and bone protective effects of mixed extracts of *P. ginseng*, *Z. jujube*, *R. fructus*, *A. asiaticae* and *S. baicalensis* (PZRAS) in vitro and in mice.

## Materials and Methods

### PZRAS Preparation Process

The plant extracts were prepared, organized and purified by Hanpoong Pham & Foods Co., Ltd. ( Korea). Briefly, *P. ginseng* was extracted with 60% ethanol while *Z. jujube*, *R. fructus*, *A. asiaticae*, and *S. baicalensis* were extracted with 30% ethanol, respectively. *P. ginseng*, *Z. jujube*, *A. asiaticae*, and *S. baicalensis* were obtained from Dong Kyung Pharm Co., Ltd. (Korea) and *R. fructus* was purchased from Omniherb Co., Ltd. (Korea). Each sample solution was separated through a 5 μm membrane filter, evaporated under reduced pressure and dried at 70-80°C. For experiment, powdered samples were weighed and mixed at a mass ratio of 4:1:1:1:1 (w/w). DMSO was used to dissolve PZRAS, which was then diluted in medium for cell culture as needed.

### Cell Culture and Osteoclast Differentiation

RAW264.7 cells from American Type Culture Collection (ATCC, USA) were cultured with Dulbeccos Modified Eagles Media (DMEM) supplemented with 10% Fetal Bovine Serum (FBS) and 100 units/ml penicillin and 100 μg/ml streptomycin at 37°C in 95% humidity and 5% CO_2_. For osteoclast differentiation, RAW264.7 cells (3 × 10^3^ cells/96 wells) were incubated with 50 ng/ml Receptor activator of nuclear factor kappa-Β ligand (RANKL) with or without PZRAS for 6 days and stained using the Tartrate-resistant acid phosphatase (TRAP) staining kit (Sigma-Aldrich, USA).

### 2,2-Diphenyl-1-Picrylhydrazyl (DPPH) Antioxidant Assay

The DPPH assay method was performed to measure antioxidant potential of PZRAS according to the manufacturer’s protocol. PZRAS was mixed with 0.1 M of DPPH in culture plates and maintained at 37°C during 30 min. DPPH radical scavenging activity of PZRAS was calculated by the following formula:

$$\mathrm{DPPH}\mathrm{radical}\mathrm{scavenging}\mathrm{activity}(\%)=[1–A_{\mathrm{sample}(540\mathrm{nm})}/A_{\mathrm{control}(540\mathrm{nm})}]\times 100$$

### MTT Assay

MTT (3-(4, 5-dimethylthiazolyl-2)-2, 5-diphenylthtrazolium bromide) was used to evaluate cell viability [29]. Cells (2 × 10^4^ cells/well) were stimulated with or without PZRAS for 24 h, and incubated with 100 μg/ml of MTT reagent in each well for 2 h further. For cell viability, the optical density (OD) of the solution was recorded at 540 nm using a microplate reader (Molecular Devices, USA).

### Lipid Peroxidation (MDA) and Enzyme Activity Assays

Thiobarbituric acid (TBA) was used to determine the amount of lipid peroxidation as previously identified [30]. The absorbance of solution was read at 532 nm and calculated based on the ε value of 153,000.

Catalase (CAT) activity was measured by Aeby’s method [31]. Superoxide dismutase (SOD) and GSH-Px activity were determined using an SOD determination kit (Oxis Research, USA) and a commercially available kit (Sigma-Aldrich) according to the manufacturers’ guidance, respectively.

### Western Blot Analysis

Western blotting was carried out by previously reported method [32]. In brief, whole cell lysates were obtained using radioimmuno-precipitatiotion assay (RIPA) buffer (iNtRON Biotechnology, Korea) containing 1 mM phenylmethylsulfonylfluoride (PMSF), and 1× protease inhibitor cocktail. Proteins were separated using SDS-PAGE and transferred subsequently onto PVDF membrane (Bio-Rad, USA) through a wet transfer system at 100 V and 350 mA. The protein bands were detected using chemiluminescence (ECL Plus Kit, Amersham Biosciences, USA) and β-actin typically was utilized as a loading control.

### Enzyme-Linked Immunosorbent Assay (ELISA)

To assess the secreted levels of pro-inflammatory cytokine (TNF-α and IL-1β), cells were pre-treated with PZRAS for 30 min and stimulated by LPS (2 μg/ml) for 8 h and 24 h, respectively. TNF-α and IL-1β ELISA kits (R&D Systems, USA) were used to measure concentrations of cytokines in culture supernatants.

### Reverse Transcription-Polymerase Chain Reaction (RT-PCR)

According to the manufacturer’s instructions, total RNA was extracted using TRI^zol^ reagent (Invitrogen, USA). SuperScript First-Strand Synthesis System (Invitrogen) was used to synthesize cDNA and the following primers were designed by Bioneer (Korea) for amplification: MMP-2, sense strand 5′-ggctggaacactaggac-3′, antisense strand 5′-cgatgccatcaaagacaatg-3′ (product size 289 bp); MMP-9, sense strand 5′-cgtcgtgatccccacttact-3′, antisense strand 5′-tcctgggcaagcagtactct-3′ (product size 433 bp); TRAP, sense strand 5′-ctgctgggcctacaaatcat-3′, antisense strand 5′-ggtagtaagggctggggaag-3′ (product size 400 bp); Cathepsin K, sense strand 5′-aggcggctatatgaccactg-3′, antisense strand 5′-ccgagccaagagagcatatc-3′ (product size 403 bp); c-Fos, sense strand 5′-atgggctctcctgtcaacac-3′, antisense strand 5′-ggctgccaaaataaactcca-3′ (product size 480 bp); NFATc1, sense strand 5′-gggtcagtgtgaccgaagat-3′, antisense strand 5′-aggtgggtgaagactgaagg-3′ (product size 280 bp); and β-actin, sense strand 5′-agaaaatctggcaccacacc-3′, antisense strand 5′-ccatctcttgctcgaagtcc-3′ (product size 435 bp). After PCR reaction, PCR products were separated using 1.5-2.0 % agarose gel electrophoresis with 0.5 × TAE buffer and confirmed by comparison to a GeneRuler 100 bp DNA Ladder Plus (Fermentas, Canada).

### Lipopolysaccharides (LPS)-Induced Bone Resorption

Animal experiments were performed under the guidance of the Jeonbuk National University Laboratory Animal Center (Approval no. CBNU 2018-094, Korea). Previous studies revealed that LPS leads to inflammatory bone resorption by direct intraperitoneal injection in mice as a bone erosion animal model [33]. Briefly, six-week-old ICR mice were injected intraperitoneally on days 1, 4, 7, 11 with or without LPS (5 mg/kg body weight). PZRAS (100, 200 μg/kg and 400 μg/kg of body weight) was administered orally 1 day prior to LPS injection and every day thereafter for 14 days. Six week-old male ICR mice used during the experiments were obtained from Damool Science (Korea).

### Microcomputed Tomographic Bone Analysis

The femurs on the left sides of the mice were scanned with a 1076 Micro CT System (Skyscan, Belgium), as previously described [34]. A three-dimensional image reconstruction analysis system was used to assess bone volume / tissue volume fraction (BV/TV, %).

### Histological Bone Analysis

Paraffin section and staining preparation were determined as described previously [32]. The femurs of the other side were processed through fixation, decalcification, embedding, sectioning, and H&E staining. Images were then obtained by a color digital video camera (ZEISS, West Germany) at 25 × and 100 × magnifications.

### Statistical Analysis

All the experiments were repeated at least three times and expressed as means ± S.D., unless otherwise indicated. Statistical analyses were performed using SPSS ver. 12.0 software, and *P*-values less than 0.05 were considered statistically significant.

## Results

### Effect of PZRAS on Cell Viability of Mouse Macrophage RAW264.7 cells

**Fig. 1:**
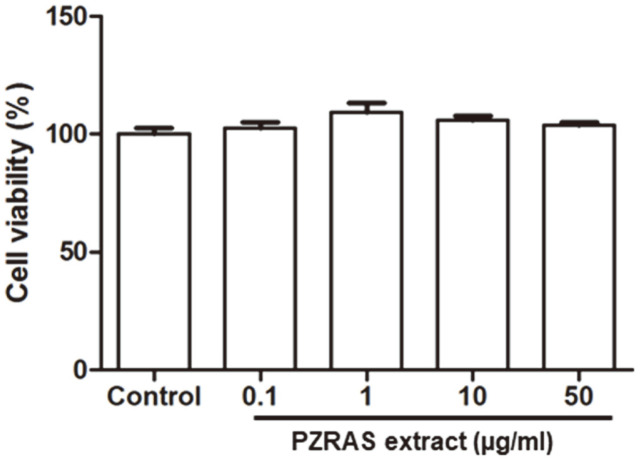
Effect of PZRAS on cell viability. RAW264.7 cells were seeded for 16 h, and then treated with various concentrations (0.1, 1, 10, and 50 μg/ml) of PZRAS for 24 h. Cell viability was determined as described in Materials and Methods. The values determined the means ± SD of at least three independent experiments.

The cytotoxicity of PZRAS was evaluated by MTT assay. RAW264.7 cells were seeded and incubated with or without PZRAS (0, 0.1, 1, 10, and 50 μg/ml) for 24 h. PZRAS showed no significant cytotoxicity compared with control group (Fig. 1).

### Effect of PZRAS on DPPH Radical Scavenging Activity

DPPH radical scavenging analysis was extensively used to measure the antioxidant effect of biological samples [35]. The radical scavenging activity of PZRAS was compared with vitamin C as the standard antioxidant. As shown in Table 1, PZRAS exhibited radical scavenging activity with an IC_50_ of 16.0 ± 1.4 μg/ml, whereas vitamin C showed 10.4 ± 2.1 μg/ml. Therefore, PZRAS has a radical scavenging activity comparable to vitamin C.

### Effects of PZRAS on Antioxidant Enzymes

**Fig. 2:**
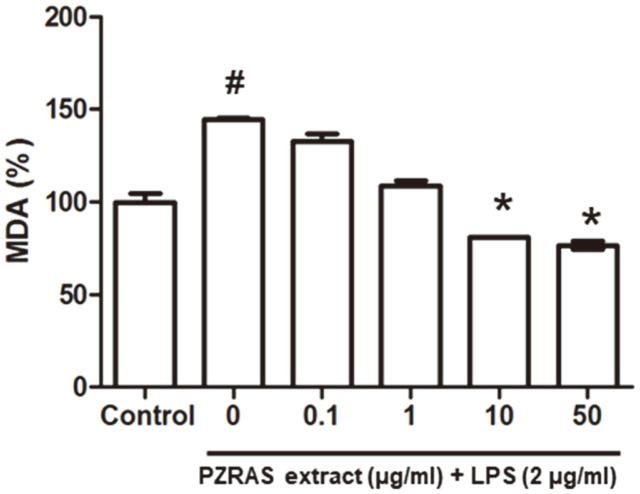
Effect of PZRAS on malondialdehyde (MDA) in RAW264.7 cells. Cells were treated with or without PZRAS for 2 h, and then incubated with LPS (2 μg/ml) for 20 h. The values determined the means ± SD of at least three independent experiments. *p <0.05 versus LPS-alone cells (#).

To confirm whether PZRAS exhibited an antioxidant activity, activities of CAT, SOD, GSH-R, and GSH-Px and MDA level were measured. As shown in Table 2, CAT activity was significantly enhanced by a 10 μg/ml concentration of PZRAS (4.43 ± 0.05 units/mg) as well as in a 50 μg/ml (4.46 ± 0.04 units/mg) concentration compared with LPS-treated group (3.90 ± 0.03 units/mg). Moreover, the activity of SOD significantly increased in 10 and 50 μg/ml PZRAS (98.0 ± 3.7 and 108.1 ± 2.8 units/mg) concentrations compared with the LPS-alone group (61.6 ± 4.8 units/mg). PZRAS treatment markedly suppressed GSH-Px and GSH-R in LPS-induced RAW264.7 cells. However, GSH-Px and GHS-R activity significantly increased in concentration of 1 μg/ml PZRAS (33.9 ± 0.92 and 566.1 ± 13.90 units/mg, respectively). The MDA level was higher than control in LPS-stimulated RAW264.7 cells, whereas PZRAS markedly reduced MDA level in 10 and 50 μg/ml concentrations compared with LPS-only group (Fig. 2). The results suggest that high doses (10 and 50 μg/ml) of PZRAS are more potent than low doses and PZRAS exhibits effective antioxidant capacity in LPS-treated RAW 264.7 cells.

### Effect of PZRAS on LPS-Induced Pro-Inflammatory Cytokines

**Fig. 3:**
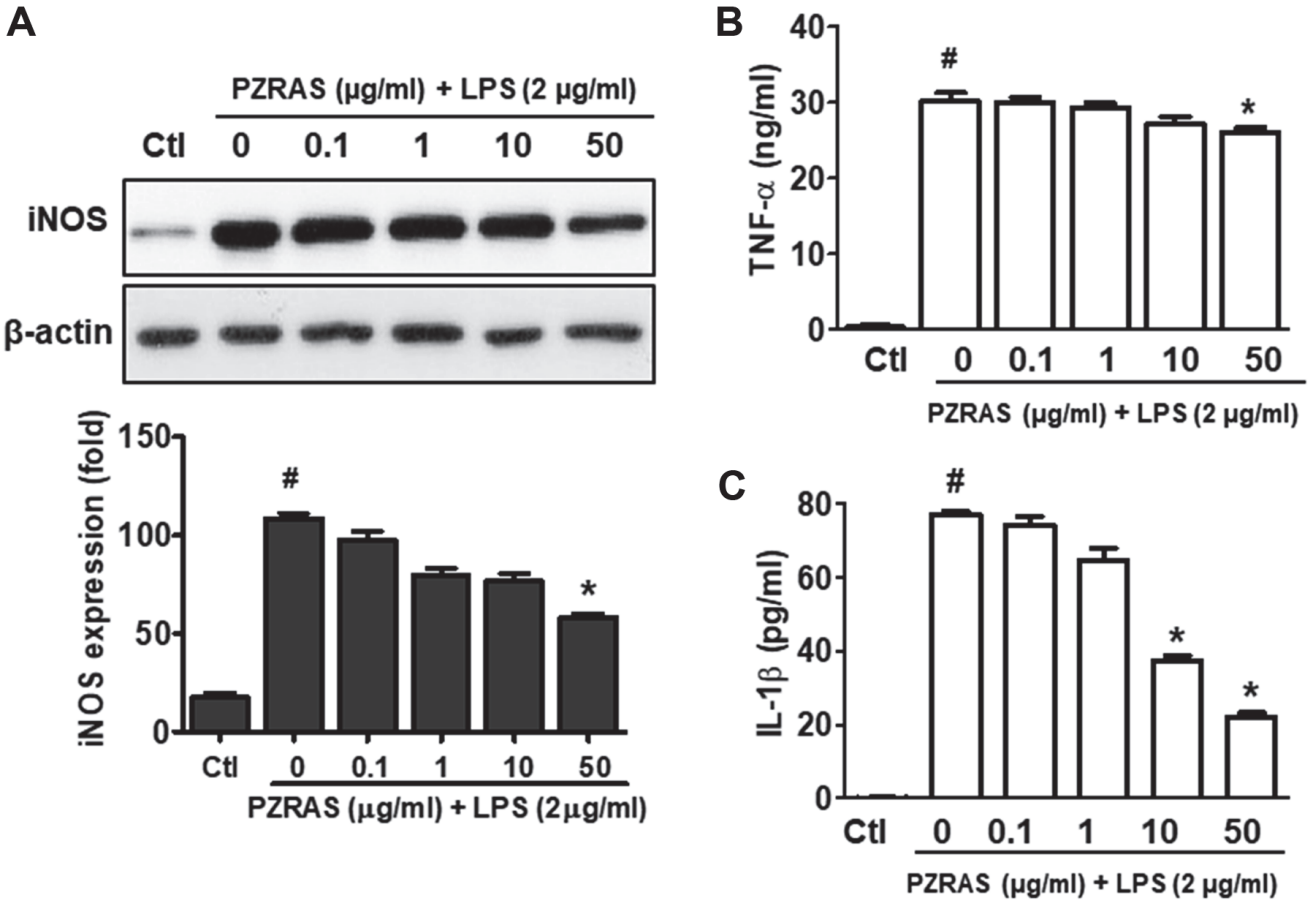
Effects of PZRAS on anti-inflammatory activities in LPS-stimulated RAW264.7 cells. Cells were pre-treated with PZRAS, and then stimulated with LPS (2 μg/ml) for 20 h. (**A**) Protein level of iNOS was assayed by western blot analysis. The secreted levels of TNF-α (**B**) and IL-1β (**C**) were assessed by commercially available ELISA kit. Data represent the means ± SD of at least three independent experiments. *p < 0.05 versus LPS-alone cells (#).

In order to explore the anti-inflammatory effect of PZRAS, RAW264.7 cells were treated with or without PZRAS. As shown in Fig. 3A, the stimulation with LPS increased the iNOS protein expression in RAW264.7 cells. However, 50 μg/ml of PZRAS downregulated LPS-induced iNOS protein level compared with LPS-only group (*p* < 0.05).

Generally, TNF-α and IL-β are released by LPS-stimulated macrophage cells and are associated with increased inflammatory responses [36]. To better perceive the effect of PZRAS on inflammation, secreted levels of TNF-α and IL-β in the cultured medium of RAW 264.7 cells were measured by ELISA. TNF-α level was markedly upregulated by LPS treatment, but PZRAS at 50 μg/ml concentration significantly decreased this induction (*p* < 0.05) (Fig. 3B). The level of IL-1β was increased by LPS treatments, and this increased level of IL-1β was strongly suppressed by PZRAS (Fig. 3C). These results indicated that PZRAS can manipulate the LPS-induced pro-inflammatory activity of cytokine, TNF-α and IL-β.

### Effects of PZRAS on RANKL-Activated Osteoclast Differentiation and mRNA Expression in RAW264.7 Cells

**Fig. 4:**
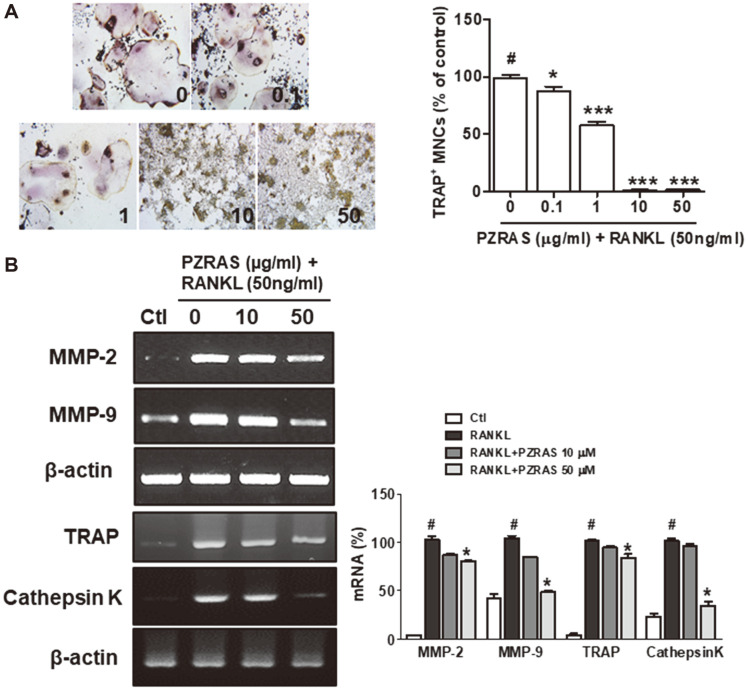
TRAP staining and RT-PCR in RANKL-induced RAW264.7 cells treated with PZRAS. Cells were maintained with indicated concentrations of PZRAS in the presence of RANKL (50 ng/ml). (**A**) After 6 days, cells were stained and photographed, and the number of TRAP-positive multinucleated (TRAP^+^ MNCs) were counted (**B**) The mRNA expressions of MMP-2, MMP-9, TRAP, and cathepsin K were analyzed by RT-PCR. The values determined the means ± SD of at least three independent experiments. *p < 0.05 versus LPS-alone cells (#).

Several pro-inflammatory cytokines including IL-1β, TNF- α, IL-6, IL-15, IL-17, and IL-18 were reported to regulate osteoclastogensis [37]. To examine the anti-osteoclast property of PZRAS, RAW264.7 cells were differentiated with RANKL and/or PZRAS. In the absence of PZRAS, RAW264.7 cells differentiated into mature TRAP-positive multinucleated cells (MNCs), but PZRAS inhibited the differentiation into matured MNCs (Fig. 4A). RANKL-RANK signaling is related with the elevation of osteoclastogenesis-related genes such as TRAP, cathepsin K, and MMP-2, -9 [38]. RT-PCR was used to verify the effects of PZRAS on RANKL-induced mRNA expression in RAW264.7 cells. The mRNA expressions of MMP-2, -9, TRAP, and cathepsin K were induced by RANKL. However, PZRAS at 50 μg/ml significantly suppressed this induction (*p* < 0.05) (Fig. 4B).

### Effects of PZRAS on RANKL-Induced MAPK Expression in RAW264.7 Cells

**Fig. 5:**
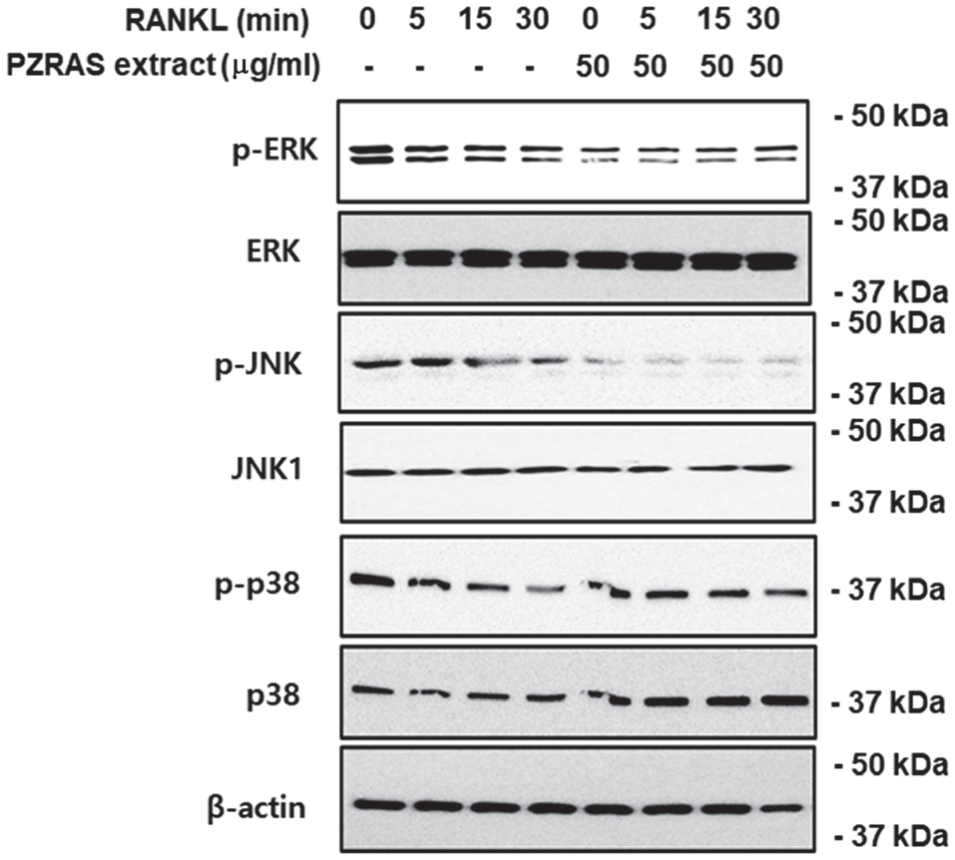
Effects of PZRAS on MAPKs in RANKL-stimulated cells. RAW264.7 cells were pretreated with 50 μg/ml of PZRAS, and induced with RANKL (50 ng/ml) for indicated times. Expressed amounts of proteins were assessed by immunoblot against specific antibodies and normalized using β-actin.

The mitogen-activated protein kinases, ERK, JNK, and p38 were stimulated through RANKL-RANK signaling pathway in osteoclast precursor cells or RAW264.7 cells [39, 40]. To clarify the intracellular mechanism of PZRAS in RANKL-signaling pathway, activities of ERK, JNK, and p38 were examined by western blot analysis. As shown in Fig. 5, RANKL at 50 ng/ml activated all three MAPKs in RAW264.7 cells, whereas PZRAS downregulated the activities of ERK and JNK but not p38 (Fig. 5).

### Effects of PZRAS on NFATc1 and c-Fos in RAW264.7 Cells

**Fig. 6:**
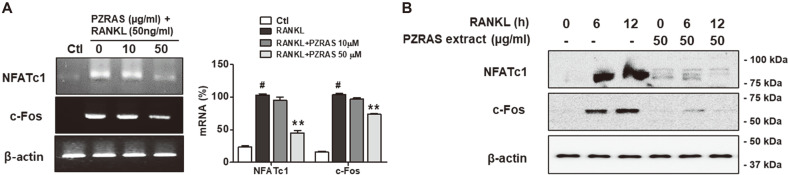
Effects of PZRAS on NFATc1 and c-Fos in RANKL-stimulated cells. RAW264.7 cells were treated with PZRAS in the presence of RANKL (50 ng/ml). (**A**) The mRNA expressions of NFATc1 and c-Fos were analyzed by RT-PCR and the histogram exhibited the levels of mRNA (%) of NFATc1 and c-Fos compared with control (#). (**B**) The protein expressions of NFATc1 and c-Fos were analyzed by immunoblot against specific antibodies. **p <0.01 versus LPS-only cells (#).

The RANKL-RANK axis resulted in the activation of RANKL-induced transcription factors such as NFATc1 or c-Fos during osteoclast formation [41, 42]. In a next step, effects of PZRAS on the expressions of NFATc1 or c-Fos were investigated during osteocalstogenesis of RAW264.7 cells. RANKL upregulated mRNA expressions of NFATc1 or c-Fos compared with control group but 50 μg/ml of PZRAS significantly reduced those transcription activities (Fig. 6A). RANKL also increased the protein expressions of NFATc1 and c-Fos within 12 h in RAW264.7 cells. However, PZRAS inhibited those protein levels compared to untreated group (Fig. 6B). These results imply that PZRAS can prevent osteoclast maturation by inhibiting RANKL-induced NFATc1 and c-Fos.

### Effect of PZRAS on LPS-Treated Bone Lysis

**Fig. 7:**
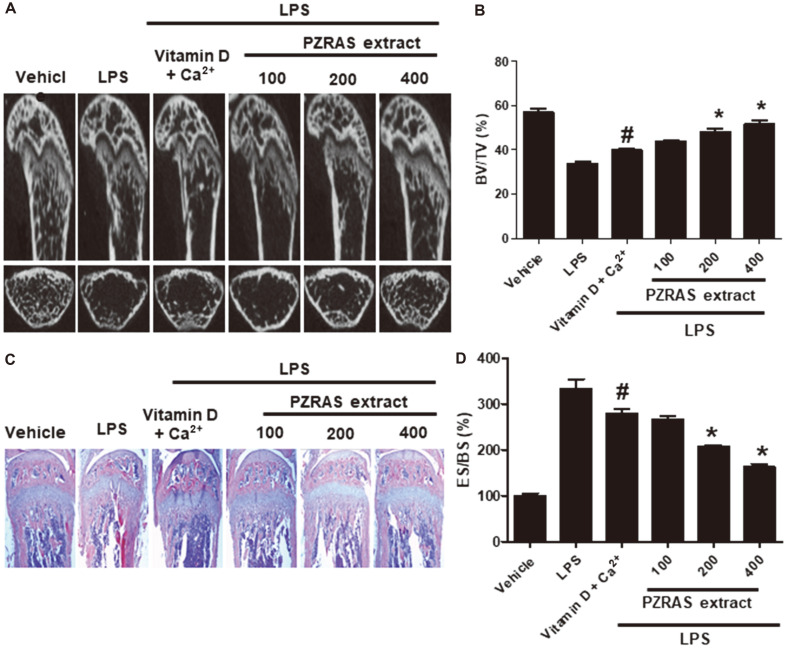
Effects of PZRAS on LPS-mediated bone erosion. (**A**) Six-week-old ICR mice were injected intraperitoneally with or without LPS (5 mg/kg body weight). PZRAS was orally administered 1 day prior to LPS injection and every day thereafter for 14 days. Tissues and organs were collected and subjected to radiographs of the longitudinal and transverse section of the proximal femurs by micro CT. (**B**) The histogram represents the bone volume/tissue volume (BV/TV) of the femurs. The vitamin D (85 mg/kg) + Ca^2+^ (1 μg/ml) group was used as a positive control. (**C**) Histological analysis of murine femurs was carried out for bone morphology and osteoclast marker was detected by H&E staining. (**D**) Eroded bone surface (BS) of the femurs near the growth plate was examined by histomorphometric analysis. *p < 0.05 versus vitamin D + Ca^2+^ (#).

To examine the suppressive effect of PZRAS on bone lysis, 6-week-old mice were injected with LPS without or with different PZRAS. We also examined the efficiency of PZRAS for bone loss by comparing with an antioxidant complex (85 mg/kg vitamin D and 1 μg/ml Ca^2+^). Micro-CT images revealed that LPS induced serious bone destruction in murine femurs. In contrast, PZRAS significantly diminished LPS-induced bone destruction at the indicated concentrations (Fig. 7A). The microstructural indices in femurs revealed that LPS injection significantly decreased bone volume/tissue volume (BV/TV) compared with vehicle group (*p* < 0.05). However, those reductions were markedly reversed by PZRAS at 200 or 400 mg/kg (Fig. 7B). H&E stain revealed that osteoclast maturation and bone loss by LPS were significantly recovered by treatment with PZRAS. Although LPS significantly caused bone loss in the femurs, PZRAS protected them from bone loss (Fig. 7C). Although the parameter of eroded bone surface per bone surface (ES/BS) of femurs significantly increased in the LPS-injected mice compared to vehicle group (*p* < 0.05), this ratio was significantly recovered in PZRAS-treated mice (*p* < 0.05)(Fig. 7D). Taken together, the above results strongly suggest that PZRAS can inhibit osteoclast differentiation and bone loss.

## Discussion

Natural flavonoid compounds are good targets of therapeutic agents because they are not associated with severe risk and possibility of long-term treatment. Therefore, the selection of natural compounds can be important to develop therapeutics for osteoporosis [7].

In this study, various compounds of flavonoids, *P. ginseng*, *Z. jujube*, *Rubi fructus*, *A. asiaticae* and *S. baicalensis* (PZRAS) were selected for anti-inflammatory activity and examined whether they had a protective effect against bone loss. Thus, we elucidated the anti-inflammatory activities of PZRAS in both in vitro and in vivo systems. The dual activities against iNOS and inflammation cytokine IL-1β were associated with increased antioxidant properties (CAT, SOD, GSH-Px and GSH-R). In addition, PZRAS has shown significant efficacy in protecting from LPS-induced bone loss, suggesting its potential therapeutic usage for bone disorders.

In previous studies, LPS can activate the productions of TNF-α, IL-1β, NO and other oxidative parameters [43, 44]. Moreover, abnormal expressions of TNF-α, IL-1β, and NO are associated with chronic inflammatory diseases [45]. Therefore, the suppression of pro-inflammatory cytokines and regulators could be applied for treating immune disorders [36]. A recent study demonstrated that *P. ginseng* leaf extract including ginsenosides Rd and Km inhibited TNF-α-enhanced expression of iNOS in HepG2 cells [46]. In this study, PZRAS dramatically repressed the expression of iNOS as well as secretion of IL-1β and TNF-α in LPS-stimulated RAW264.7 cells. These results suggest that PZRAS may exhibit the anti-inflammatory capacity through suppression of pro-inflammatory factors such as iNOS, IL-1β or TNF-α.

Free radical attack on plasma membrane components such as LPS generates MDA. GSH is a strong antioxidant for cellular detoxification processes which can reduce MDA production. Therefore, it has been suggested that endogenous GSH plays an essential role in preventing oxidative stress-related inflammation [13]. In this study, PZRAS effectively increased activities of CAT, SOD, and GSH-Px and markedly decreased the MDA level (Table 2 and Fig. 2). These results suggest that PZRAS may act as an antioxidant agent via upregulation of CAT, SOD, and GSH-Px activities and suppression of MDA production.

Previous studies have demonstrated that the RANK/RANKL axis leads to activation of several downstream signaling pathways for osteoclast-specific genes and transcription factors [38-40]. For instance, MAPKs regulate NFATc1 and c-Fos, transcription factors which then modulate the MMP-9 mRNA expression during osteoclast maturation [41, 42]. PZRAS significantly inhibited phosphorylated ERK and JNK, (but not p38 expression), mRNA expressions of MMP-2, -9, TRAP, cathepsin K as well as transcription factors (NFATc1, c-Fos) during osteoclast differentiation. We also demonstrated that PZRAS efficiently worked to cure bone loss in LPS-induced animal model as evidenced by its improving BV/TV and ES/BS.

The above results clearly show that PZRAS exhibits an anti-osteoclastogenic potential by reducing the generation of iNOS, IL-1β, and MMP-2, -9, and also increased levels of antioxidant enzymes (CAT, SOD, GSH-Px and GSH reductase). PZRAS also prevented LPS-induced bone destruction in mice. Therefore, our findings suggest that PZRAS could be a promising therapeutic candidate for various bone-related diseases.

## Figures and Tables

**Table 1:** Radical scavenging activities of PZRAS and vitamin C.

	IC_50_ (μg/ml)
PZRAS	16.0 ± 1.4
Vitamin C	10.4 ± 2.1

**Table 2:** Effects of PZRAS on antioxidant enzyme activities in LPS-induced RAW264.7 cells.

	Untreated		PZRAS (μg/ml) + LPS (2 μg/ml)			

		0	0.1	1	10	50
^a^ CAT (Units/mg Protein)	3.78 ± 0.80	3.90 ± 0.03^#^	3.88 ± 0.02	4.01 ± 0.04	4.43 ± 0.05^*^	4.46 ± 0.04^*^
^b^SOD (Units/mg Protein)	46.9 ± 1.90	61.6 ± 4.80^#^	65.7 ± 7.40	77.9 ± 3.60	98.0 ± 3.70^*^	108.1 ±2.80^*^
^c^ GSH-Px (mU/ml Protein)	36.14 ± 0.49	25.3 ± 0.44	28.84 ± 1.00	33.9 ± 0.92^*^	26.72 ± 0.13	19.36 ± 0.28
^d^ GSH-R (μmol/L Protein)	622.9 ± 24.80	448.5 ± 6.60^#^	520.2 ± 28.50	566.1 ± 13.90^*^	420.3 ± 20.10	390.1 ± 25.20

^#^*p* < 0.05 as compared with the untreated group. ^*^p < 0.05 as compared with the LPS-treated group only.

a. catalase; b. superoxide dismutase; c. glutathione peroxidase; d. glutathione reductase
